# Micro-Raman Vibrational Identification of 10-MDP Bond to Zirconia and Shear Bond Strength Analysis

**DOI:** 10.1155/2017/8756396

**Published:** 2017-10-02

**Authors:** Diego Martins De-Paula, Alessandro D. Loguercio, Alessandra Reis, Natasha Marques Frota, Radamés Melo, Kumiko Yoshihara, Victor Pinheiro Feitosa

**Affiliations:** ^1^Federal University of Ceará, Fortaleza, CE, Brazil; ^2^Universidade Estadual de Ponta Grossa, Ponta Grossa, PR, Brazil; ^3^Paulo Picanço School of Dentistry, Fortaleza, CE, Brazil; ^4^Center for Innovative Clinical Medicine, Okayama University Hospital, Okayama, Japan

## Abstract

So far, there is no report regarding the micro-Raman vibrational fingerprint of the bonds between 10-methacryloyloxy-decyl dihydrogen phosphate (10-MDP) and zirconia ceramics. Thus, the aim of this study was to identify the Raman vibrational peaks related to the bonds of 10-MDP with zirconia, as well as the influence on microshear bond strength. Micro-Raman spectroscopy was employed to assess the vibrational peak of 10-MDP binding to zirconia. Microshear bond strength of the dual-cure resin cement to zirconia with the presence of 10-MDP in composition of experimental ceramic primer and self-adhesive resin cement was also surveyed. Statistical analysis was performed by one-way ANOVA and Tukey's test (*p* < 0.05). Peaks at 1545 cm^−1^ and 1562 cm^−1^ were found to refer to zirconia binding with 10-MDP. The presence of 10-MDP in both experimental ceramic primer and self-adhesive resin cement improved microshear bond strength to zirconia ceramic. It can be concluded that the nondestructive method of micro-Raman spectroscopy was able to characterize chemical bonds of 10-MDP with zirconia, which improves the bond strengths of resin cement.

## 1. Introduction

Highly crystalline ceramics based on zirconium oxide have been applied for decades in Restorative Dentistry, providing successful rehabilitation. Its principal characteristics are the great mechanical properties such as high flexural strength (900–1200 MPa), fracture toughness (9-10 MPa·m^1/2^), compression strength (~2000 MPa), and Young's modulus (100–210 GPa) as well as adequate aesthetics and biocompatibility. Overall, these features make zirconia the ideal material for core and frameworks of prosthesis in anterior and posterior region [1–3].

The large content of polycrystals (up to 99.9%) affords chemical stability to zirconia, thereby reducing the reactivity with acids (even hydrofluoric acid) [1]. Indeed, one of the major shortcomings of zirconia's usage is the difficulty to bond using resin-based cements and the luting on dental substrate [4]. In a recent systematic review, Inokoshi et al. [5] concluded that most effective and durable protocol for adhesion to zirconia ceramics involves the pretreatment with silica air-abrasion followed by application of primers containing silane/10-MDP (10-methacryloyloxy-decyl-dihydrogenphosphate) before using dual-cure resin cements.

Acidic functional monomer 10-MDP is currently considered as gold standard in terms of chemical bonding and clinical longevity [6, 7] thanks to the unique chemical structure with long and hydrophobic spacer carbon chain [8, 9]. The rationale for employing a 10-MDP primer on zirconia surface is to create a “reactive” surface, favouring the binding of 10-MDP phosphate functionalities with zirconium oxide [10]. Unfortunately, the characterization of 10-MDP bonds has been performed by transmission electron microscopy [11, 12] or nuclear magnetic resonance [13], which are time-consuming and laborious methodologies. However, to the extent of our knowledge, there are no reports in literature regarding the identification of such chemical bond between 10-MDP and zirconia by means of micro-Raman vibrational spectroscopy, as well as the improvements afforded by such bonding on the bond strengths of resin cements to zirconia substrate.

Therefore, the objective of this study was to assess the Raman peak related to the bond of 10-MDP to zirconia. Also, further aim was to survey the bond strengths to zirconia when 10-MDP is used in a ceramic primer or in self-adhesive resin cement. Study hypothesis is that the presence of 10-MDP bond to zirconia does ameliorate the bond strength of resin cement.

## 2. Materials and Methods

### 2.1. Experimental Design

The present study is composed of two parts: (1) evaluation of Raman peak related to the bond of 10-MDP to zirconia and microshear bond strength to zirconia varied ceramic primer and (2) evaluation of Raman peak related to the bond of 10-MDP to zirconia and microshear bond strength to zirconia varied self-adhesive resin cement. For the first purpose, three groups were tested: a commercial primer (CP; Clearfil Ceramic Primer; Kuraray Medical Inc., Tokyo, Japan), an experimental primer (EP), and a negative control group (NP, no primer). To formulate the experimental primer, 20 wt% 10-MDP was mixed with 40 wt% distilled water and 40 wt% absolute ethanol. For the second purpose, also three groups were evaluated: a commercial self-adhesive resin cement RelyX U200 (CCP; 3M ESPE; St. Paul, MN, USA), an experimental self-adhesive resin cement (ECP) with the presence of MDP, and an experimental 10-MDP-free resin cement (ECN) without the addition of 10-MDP or other acidic functional monomer.

The experimental resin cements were prepared in two pastes in order to avoid chemically initiated polymerization and preionization of acidic monomers. The composition of each paste was as follows.

Experimental ECP: Paste A: BisGMA (20 wt%), TEGDMA (20 wt%), fillers (50 wt%), 10-MDP (10 wt%), CQ (1 wt%), benzoyl peroxide (1 wt%) and; Paste B: BisGMA (20 wt%), TEGDMA (20 wt%), fillers (50 wt%), 10-MDP (10 wt%), 2% EDAB (2 wt%).

Experimental ECN: Paste A: BisGMA (25 wt%), TEGDMA (25 wt%), fillers (50 wt%), CQ (1 wt%), benzoyl peroxide (1 wt%) and; Paste B: BisGMA (20 wt%), TEGDMA (20 wt%), fillers (50 wt%), 2% EDAB (2 wt%).

For manipulating all experimental products, the following chemical agents were used: monomers 2,2-bis[4-(2-hydroxy-3-methacryloxyprop-1-oxy)phenyl]propane (Bis-GMA) and triethylene-glycoldimethacrylate (TEGDMA) donated by Esstech Inc. (Essington, PA, USA), used as received. Acidic monomer 10-MDP (10-methacryloyloxy-decyl-dihydrogen-phosphate) was donated by FGM (Joinville, Brazil). Photoinitiator system was composed of camphorquinone (CQ, Esstech), chemical initiator benzoyl peroxide (Sigma Aldrich, St. Louis, USA), and coinitiator ethyl 4-(dimethylamino)benzoate (EDAB, Sigma Aldrich). Silanated barium borosilicate glass particles (0.4 *μ*m average size, Esstech) were used as filler particles. Commercial materials used in the present study are listed in Table 1.

### 2.2. Specimen Preparation

Forty-two yttria-stabilized tetragonal zirconia polycrystal (Y-TZP) ceramic blocks (Zirconcad, Angelus, Londrina, Brazil) with 13.2 × 13.2 × 3.2 mm dimensions were obtained and sintered according to manufacturers' instructions. They were embedded and fixed in acrylic resin (Jet Classico Ltda., Campo Limpo Paulista, Brazil). The exposed flat zirconia surfaces were polished for 30 s with 600-, 800- and 1200-grit SiC papers under water irrigation and then ultrasonicated for 10 minutes.

### 2.3. Micro Shear Bond Strength Evaluation

Following, eighteen Y-TZP blocks were divided into three groups. 10-MDP containing ceramic primers were applied onto Y-TZP surfaces (*n* = 6 for group). Either experimental primer (EP) or the commercial primer (CP) was actively applied for 20 s followed by a gentle air-blast for 5 s. In negative control group, no primer was used before resin cement bonding.

Microshear bond strength (*μ*-SBS) specimens were bonded to the zirconia surfaces using cylindrical translucent moulds (Tygon tubing, TYG-030; Saint-Gobain Performance Plastic, Clearwater, USA) as previously reported [14]. Six cylinders (0.75 mm diameter × 3 mm height) were randomly bonded for each group (*n* = 6) using the dual-cure resin cement RelyX ARC (3M-ESPE, St. Paul, MN, USA).

For the second purpose, eighteen Y-TZP blocks were prepared as previously described and they were divided into three groups: bonded with the experimental self-adhesive resin cement (ECP), bonded with the commercial cement (CC) RelyX U200 (3M-ESPE), and the control resin cement (ECN) group. Resin cements were manipulated according to the manufacturer's instructions using the same volume of each paste and mixing for 30 s until obtaining homogeneous mixture. The experimental cements also followed this protocol, and then cements were carefully inserted in the Tygon tubes to avoid blisters.

All specimens for both groups were light-cured with LED light-curing unit DB-685 (1100 mW/cm^2^; Dabi Atlante, Ribeirão Preto, Brazil) for 40 s. After 30 minutes, each Tygon tube was removed with scalpel blades. Cylinders were analysed by stereomicroscopy and those with defects were discarded and replaced. Before bond strength test, all specimens were immersed in distilled water at 37°C for 48 h to wait for the chemical cure of the resin cement.

Bonded specimens were mounted in a device for *μ*-SBS test (Odeme Dental Research, Luzerna, Brazil) adapted in a universal testing machine (EMIC DL 2000, Sao Jose dos Pinhais, Brazil). An orthodontic wire (0.4 mm diameter) was positioned surrounding and in contact with half of the cylinder and connected to the load cell (50N) of the machine to exert shear force in upward direction. Each cylinder was tested individually with 1 mm/min crosshead speed up to fracture. Maximum *μ*-SBS was recorded in N and transformed to MPa by dividing the results in N by the cross-sectional area of each specimen.

After debonding, all zirconia surfaces were examined with a stereomicroscope (SMZ800, Nikon, Tokyo, Japan) to determine the mode of failure that was classified into three types: A, adhesive fracture between ceramic and cement without signs of residual cement on zirconia surface; C, cohesive failure of the cement with full area presenting cement remnants; M, mixed fracture with areas depicting adhesive debonding and some residual cement indicating partial cohesive failure. Microshear bond strength outcomes for each study were statistically analysed by Kolmogorov-Smirnov normality test (*α* = 0.05), one-way ANOVA, and Tukey's test (*α* = 0.05).

### 2.4. Micro-Raman Spectroscopy Evaluation

To obtain the vibrational analysis of the bonds between 10-MDP and zirconia, the micro-Raman spectrophotometer (Xplora, Horiba Jobin Yvon, Paris, France) was calibrated internally in zero using the silicon standard sample provided by the manufacturer. The configurations of the equipment were HeNe laser with 3.2 mW power, 633 nm laser wavelength, 10 s acquisition time, 3 accumulations, 1.5 *μ*m spatial resolution, 2.5 cm^−1^ spectral resolution, 10x magnification lens (Olympus, London, UK), and 60 × 70 *μ*m field area.

For observation of Raman peak referring to the bond of zirconia with phosphate functionality of 10-MDP, three Y-TZP blocks were used for group. From each block, three initial readings (Y-TZP) were performed. Afterwards, the 10-MDP primers were applied as aforementioned for 20 s. After 10 minutes, the primed blocks were thoroughly rinsed with absolute ethanol and dried with air-blast to remove unbound monomer. Further three readings per group (*n* = 3) were conducted in experimental primer-treated samples (Y-TZP + EP) and those treated with commercial primer (Y-TZP + CP). Initially, the spectral range was set from 100 to 4000 cm^−1^. Following initial observations, the range was narrowed to 1500–2000 cm^−1^, region that depicted differences between spectra. The chemical shift and intensity of each peak were processed for baseline correction and normalized.

## 3. Results

**Figure 1:**
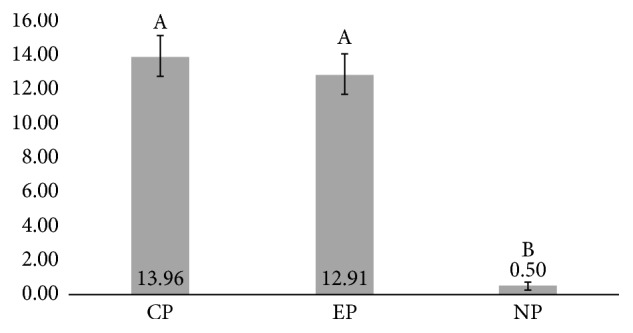
Mean and standard deviation of *μ*SBS (MPa) of dual-cure resin cement after ceramic primers (CP: Clearfil Ceramic Primer; EP: an experimental primer; NP: no primer) applied on Y-TZP ceramics. Different capital letters indicate statistical difference (*p* < 0.05).

The one-way ANOVA showed statistical significant difference between ceramic primer groups (*p* < 0.001). The results showed that the presence of 10-MDP in ceramic primer applied previously to the dual-cure resin cement increased the *μ*-SBS to Y-TZP for both experimental primer and commercial one when compared to negative control (Figure 1). The former did not show any difference between experimental and commercial primers. The failure pattern analysis showed predominantly mixed failure pattern for experimental and commercial primers, whilst adhesive fractures were most frequent in negative control.

**Figure 2:**
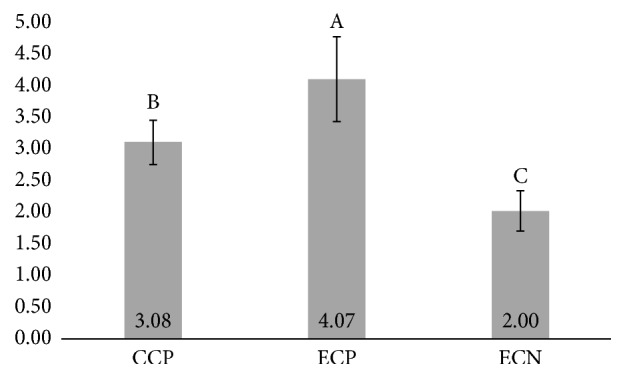
Results of *μ*-SBS test (MPa) of self-adhesive resin cements bonded to Y-TZP ceramics. (CCP: RelyX U200; ECP: experimental self-adhesive dual-cure resin cement with MDP; and ECN: experimental self-adhesive dual-cure resin cement without MDP). Different capital letters indicate significant difference (*p* < 0.05).

The one-way ANOVA showed statistical significant difference between resin cement groups (*p* < 0.001). The presence of 10-MDP as a component of self-adhesive resin cement showed statistically significant higher mean *μ*SBS when compared with other cements (Figure 2). Also, the commercial cement showed intermediary mean *μ*SBS and statistically significant higher than experimental cement without MDP, as presented in Figure 2. The failure pattern analysis depicted adhesive failures for all specimens of self-adhesive resin cements.

**Figure 3:**
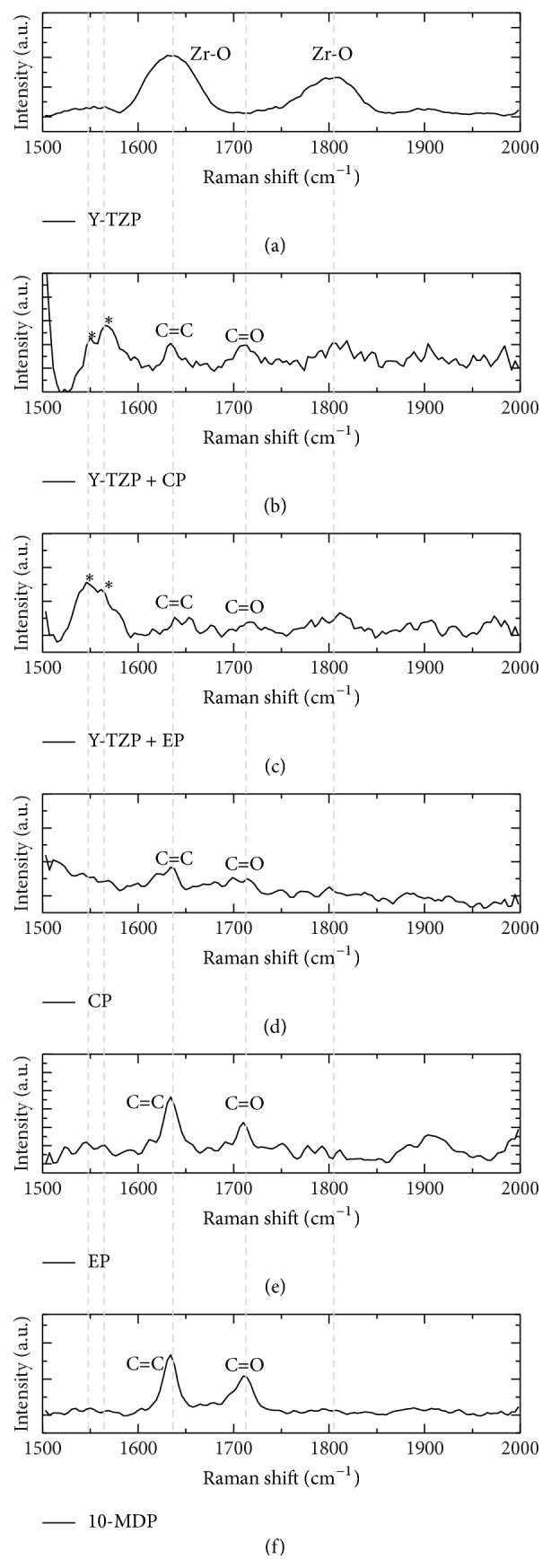
Micro-Raman spectra of Y-TZP ceramic (Y-TZP), primers-treated ceramics (Y-TZP + CP and Y-TZP + EP), commercial primer (CP), experimental primer (EP), and neat 10-MDP (10-MDP). Peaks at 1636 cm^−1^ and 1803 cm^−1^ refer to Zr-O bonds; peak at 1630cm^−1^ is relative to C=C bonds and 1710 cm^−1^ to C=O bonds of 10-MDP. Asterisks (*∗*) are indicating 1545 cm^−1^ and 1562 cm^−1^ peaks which only appeared after application of 10-MDP containing primers on Y-TZP ceramics.

Micro-Raman spectra MDP-zirconia bond are presented in Figure 3. Peaks 1636 cm^−1^ and 1803 cm^−1^ are referring to Zr-O bonds of Y-TZP. In Figures 3(d), 3(e), and 3(f), it was possible to verify only peaks at 1630 cm^−1^ and 1710 cm^−1^ from C=C e C=O vibrations of 10-MDP, respectively. Figures 3(b) and 3(c) were obtained from Y-TZP after ceramic primers application and showed less evidence (lower height) of 1636 cm^−1^ and 1803 cm^−1^ peaks (Zr-O) and presence of 1630 cm^−1^ and 1710 cm^−1^ peaks from 10-MDP. However, a wide peak was observed between 1545 cm^−1^ and 1562 cm^−1^, which was not detected in pure 10-MDP and Y-TZP alone. Therefore, it is suggested that these wide peaks are referring to coordinates bond between 10-MDP phosphate functionality and zirconia ceramic.

## 4. Discussion

In the present investigation, 10-MDP was employed as chemical method to improve the bonding of resin cement to Y-TZP, which actually occurred when included either in ceramic primers and in self-adhesive resin cement, increasing statistically the bond strength to zirconia ceramic. Therefore, the study hypothesis needs to be accepted. In this study, the micro-Raman spectroscopy was also used to find the peak of this bond interaction.

Currently, zirconia frameworks for dental prosthesis are well-established [15, 16] due to their optimal mechanical and biological properties [1, 17]. With atmospheric pressure, pure zirconia presents three crystallographic conformations, depending on the temperature. Below 1170°C, crystal structure is monoclinic (m), between 1170°C and 2370°C tetragonal (t) conformation is presented, and above 2370°C up to melting point the structure is cubic. For dental purposes, zirconia structure may be stabilized in tetragonal conformation in ambient temperature by yttrium oxide (Y_2_O_3_), the so-called Y-TZP. This augments fracture toughness, once there is phase transformation (t → m) when a crack or microcrack appears. Such transformation is followed by volume increase (3–5%) sufficient to reduce crack propagation [2, 18, 19].

In metal-free indirect restorations, feldspathic ceramic is applied in high temperatures as covering to zirconia frameworks [20]. Residual thermal stresses might be formed in zirconia-porcelain interface during cooling, which increases the likelihood of crack formation/propagation under chewing [21]. Zhang and Kim [22] developed a graded zirconia (glass/zirconia/glass) by infiltration of silica-rich ceramic. Indeed, such structure improves the aesthetics (external glass) as well as luting (silanization of internal glass ceramic) [23–25].

However, all these procedures change zirconia in a difficulty substrate to be bonding using resin-based cements and the luting on dental substrate [4], once traditional hydrofluoric acid-etching and silanization are only successful for silica-containing ceramics. Surface grinding is an alternative to enhance the roughness and micromechanical interlocking of resin cement [26]. This procedure may be achieved with abrasive papers (SiC ou Al_2_O_3_), alumina air-abrasion, and diamond burs in high-speed handpieces [27]. Thermal treatment (1200°C/2 h) is also a choice after adjustment in absence of monoclinic phase [2]. Tribochemical silica coating of internal zirconia and alumina ceramic crowns is a common procedure favouring increase of roughness along with deposition of silica to subsequent silanization [10].

10-MDP incorporation in ceramic primers or self-adhesive resin cements improves effectively bonding to zirconia [26, 28] by the chemical bonding (ionic and hydrogen bonds) of phosphate functionalities of 10-MDP and zirconium dioxide [10]. The broadening of the peaks (Figure 3) observed when zirconia was in contact with 10-MDP suggests a slight formation of amorphous zirconia crystals during the binding to the acidic monomer. Indeed, mild etching promoted by the primer on the surface of highly crystalline zirconia ceramic might have induced very superficial softening and transformation to less crystalline structure, with minor alterations on the mechanical properties of zirconia prosthesis. The acidic functional monomer is a feasible, simple, and cost-effective strategy for zirconia bonds that may be applied alone or associated with silane (for instance, in Clearfil Ceramic Primer) to be used for all types of dental ceramics [5, 27, 29].

Functional monomer 10-MDP is nowadays one of the most efficient functional monomers in terms of chemical interaction to calcium [6] providing clinical longevity [7]. Several companies have started to add 10-MDP to their products after their patent was expired [28]. Its long and hydrophobic carbon chain promotes stability for MDP-Ca salts formed with low solubility [11]. This is particularly correlated also for metallic oxides such as zirconia [10]. Methacrylate group accomplishes the copolymerization with further monomers in adhesives and resin cements whereas its hydrophobic feature warrants low hydrolytic degradation as previously demonstrated [6, 8, 13].

The “reactive” surface of Y-TZP generated by 10-MDP occurs due to the bifunctional characteristic of the monomer that bonds zirconia and resin matrix. In the present investigation, this was depicted by the increase of *μ*-SBS, in presence of 10-MDP either in ceramic primer or in self-adhesive resin cement. The present study also showed that the presence of 10-MDP in the primer is essential for attaining superior bond strength compared to using 10-MDP-containing self-adhesive resin cement (Figures 1 and 2). Herein, no method to increase the surface roughness of zirconia was employed, which is not usual in the literature [5], which suggests the combination of chemical agents (10-MDP containing primers) with alumina air-abrasion as gold-standard strategy [26]. The evidence of such chemical bond is highly relevant towards the design of new (and better) acidic functional monomers. Some analytical methods proved this bonding mechanism (10-MDP/zirconia), such as photoelectric X-ray spectroscopy (XPS) [30], Fourier transform infrared spectroscopy (FTIR) [31], and mass spectroscopy [32], but no reports were found regarding micro-Raman spectroscopy, although it is an established method for chemical characterization in dentistry [33].

Micro-Raman has some advantages like nondestructive sample preparation, analysis of liquids, powders, and no need for previous preparations. It utilizes the inelastic dispersion of light from a visible laser near to infrared or ultraviolet range in order to yield vibrational information of chemical bonds [33]. In this study (Figure 3), peaks at 1636 cm^−1^ and 1803 cm^−1^ refer to Zr–O bonds after analysis at zirconia block. Peak at 1630 cm^−1^ is relative to C=C bonds and 1710 cm^−1^ to C=O bonds of 10-MDP that were found at neat 10-MDP, commercial and experimental primers, and Y-TZP ± CP and Y-TZP ± EP proving the presence of 10-MDP onto zirconia. Raman peaks of Y-TZP (1636 cm^−1^ and 1803 cm^−1^) decreased and a wide shoulder appeared with peaks at 1545 cm^−1^ and 1562 cm^−1^. These peaks were not found in commercial primer, neat 10-MDP, and experimental primer. It is suggested that these new peaks are related to –P–O–Zr bonds, likely representing simple and double coordinate bonds showed by Xie et al. (2015) [10]. In a recent study, Xie et al., 2016 [34], also showed by XPS spectra that the peak representing zirconia ceramic is divided into two peaks after application of 10-MDP-containing primers. Future works should focus on the in situ identification of these bonds.

## 5. Conclusion

It is worth concluding that micro-Raman identification of bonds formed between 10-MDP and Y-TZP attained the formation of peaks 1545 cm^−1^ and 1562 cm^−1^. The presence of 10-MDP in primer and self-adhesive resin cement increases the microshear bond strength to Y-TZP ceramic.

## Figures and Tables

**Table 1:** Spreading of materials' composition and manufacturers.

Materials	Composition	Manufacturer
Zirconcad	99.0% ZrO_2_ + Y_2_O_3_ 0.5% Y_2_O_3_	Angelus, Londrina, Brazil

RelyX ARC	Paste A: Bis-GMA, TEGDMA, silane treated silica, functionalized dimethacrylate polymer, 2-benzotriazolyl-4-methylphenol, 4-(dimethylamino)-benzeneethanol. Paste B: silane treated ceramic, TEGDMA, Bis-GMA, silane treated silica, functionalized dimethacrylate polymer, 2-benzotriazolyl-4-methylphenol, benzoyl peroxide (72/wt).	3M ESPE, St. Paul, USA

RelyX U200	Base: Methacrylate monomers containing phosphoric acid groups, methacrylate monomers, initiators, stabilizers, rheological additives. Catalyst: Methacrylate monomers, alkaline fillers, silanated fillers, initiator components, stabilizers, pigments, rheological additives. Zirconia/silica fillers	3M ESPE, St. Paul, USA

Clearfil Ceramic Primer	10-Methacryloyloxydecyl dihydrogen phosphate, 3-(trimethoxysilyl)propyl methacrylate, ethanol	Kuraray Medical Inc., Tokyo, Japan
